# Exploration of Predictors for Korean Teacher Job Satisfaction via a Machine Learning Technique, Group Mnet

**DOI:** 10.3389/fpsyg.2020.00441

**Published:** 2020-03-13

**Authors:** Jin Eun Yoo, Minjeong Rho

**Affiliations:** Department of Education, Korea National University of Education, Cheongju-si, South Korea

**Keywords:** teacher job satisfaction, machine learning, penalized regression, Mnet, TALIS

## Abstract

Despite the high academic achievements of Korean students in international comparison studies, their teachers’ job satisfaction remains below the Organization for Economic Co-operation and Development (OECD) average. As job satisfaction is one of the major factors affecting student achievement as well as student and teacher retention, the identification of the most important satisfaction predictors is crucial. The current study analyzed data from the OECD 2013 Teaching and Learning International Survey (TALIS) via machine learning. In particular, group Mnet (a penalized regression method) was employed in order to consider hundreds of TALIS predictors in one statistical model. Specifically, this study repeated 100 times of variable selection after random data-splitting as well as cross-validation, and presented predictors selected 50% of the time or more. As a result, 18 predictors were identified out of 558, including variables relating to collaborative school climates and teacher self-efficacy, which was consistent with previous research. Newly found variables to teacher job satisfaction included items about teacher feedback, participatory school climates, and perceived barriers to professional development. Suggestions and future research topics are discussed.

## Introduction

Korea has consistently been one of the top-ranked countries in terms of students’ academic achievement in international comparison studies, including Trends in International Mathematics and Science Study (TIMSS) and the Program for International Student Assessment (PISA). Confucianism, Korea’s overarching ideology for centuries, appears to have contributed to its students’ high achievement. In countries with Confucian traditions, hard work and study are considered great virtues (Chou et al., 2013). Teachers are granted elevated social status, while students are expected to respect and obey their teachers (Tan, 2017). An old Korean proverb, “Do not even step on the shadow of a teacher,” is one example of how teachers are respected as authority figures. Despite Korea’s successful economic transformation to industrialization in the late 20th century and updates to its list of preferred occupations, the teaching profession remains one of most respected occupations in terms of social recognition and job stability. Many high scorers in the College Scholastic Ability Test (CSAT), Korea’s college entrance exam, apply to become education majors. Consequently, the teacher licensing examination has become increasingly competitive.

When these factors are taken together, one would assume that Korean teachers should be satisfied with their occupation, feel confident, and be proud to be teachers. Unfortunately, this does not seem to be reflected in the statistics. According to the 2013 Teaching and Learning International Survey (TALIS), both job satisfaction and self-efficacy of Korean teachers were below OECD averages. Although Korean teachers generally agreed that teaching is valued in the society and that the advantages of being a teacher outweigh the disadvantages, 20% regretted becoming teachers, the highest recorded percentage among all participating countries. Although Korean students have shown high academic performance in international comparison studies, the low morale of Korean teachers may create serious problems for the Korean education system in the near future because job satisfaction of teachers is one of the most influential factors that affect student achievement (OECD, 2014) as well as student and teacher retention (OECD, 2014; Sims, 2017). Therefore, the identification of teacher job satisfaction predictors is critical in order to establish methods for helping teachers regain or maintain their job satisfaction for the betterment of the teachers themselves as well as their students and the Korean society.

The current study sought to identify the most important predictors of teacher job satisfaction using the Korean teacher and principal data from TALIS 2013. Developed by OECD to provide participating countries with important indicators for effective teaching and learning, TALIS offers hundreds of variables based on the responses of thousands of teachers and hundreds of principals per country. TALIS teacher and principal questionnaires encompass various domains, from demographics, professional development (PD), and instruction quality to teacher appraisals, school management, and institutional resources. However, previous research utilizing TALIS data has focused on only a few variables selected from existing theories and research. Although this practice has been a long-standing tradition in the field of social sciences, modeling with pre-selected variables that are frequently statistically significant can hinder studies attempting to explore new variables that have not been investigated in previous research. Therefore, this study considered all possible variables provided by TALIS in one statistical model in order to explore and identify important predictors of Korean teachers’ job satisfaction via machine learning.

Machine learning was employed for the following reasons. Firstly, conventional statistical techniques including structural equation modeling (SEM) and hierarchical linear modeling (HLM) encounter difficulties when handling hundreds of variables in one statistical model, and they are likely to yield non-convergence. Machine-learning techniques are much more versatile. While conventional methods only deal with structured *long* data (i.e., more observations than variables), machine-learning techniques can handle unstructured data such as video files as well as *wide* data (i.e., more variables than observations) without encountering convergence problems (Bzdok et al., 2018, p. 233; Yoo, 2018). Secondly, unlike conventional techniques which focus on explanations, prediction is the operative word in machine learning. Conventional techniques build models that can explain current data very well, but they are less likely to consider predictability. In contrast, a model from machine learning is built to fit new data well and thus called as a predictive model. While so-called black-box methods such as support vector machines and deep learning, focusing on predictability, are known to be notoriously difficult to interpret, penalized regression produces relatively more interpretable models among machine learning techniques. Thirdly and relatedly, overfitting limits generalization and thus predictability, and machine learning prevents overfitting (Bzdok et al., 2018; Yoo, 2019). Overfitting occurs in unnecessarily complex models, particularly when the model emphasizes explanation of the current data. In order to accurately explain the current data, that data’s eccentricities may be picked up in the modeling process. This results in the inclusion of unnecessary terms only relevant to the current data in the final model, which is called overfitting. Penalized regression is employed as a regularization method to prevent overfitting in statistics.

### Literature Review

OECD (2014) defines job satisfaction as the sense of fulfillment and gratification from working in a specific occupation. In TALIS, teacher job satisfaction comprises satisfaction with the profession, including the role and work of a teacher, as well as satisfaction with the school environment. Variables representing different aspects of job satisfaction of Korean teachers were categorized as teacher or school characteristics, with the latter including variables associated with school demographics and school climate.

In particular, variables related to school climate tended to have different names in various studies even though they measured the same or similar constructs in varying degrees. Variables regarding principal leadership and teacher cooperation were indicative of school climate. Teacher empowerment was one type of principal leadership, and the school community culture or sense of community was one way to measure teacher cooperation. Variables related to PD were also categorized as teacher cooperation (Garet et al., 2001; Jurasaite-Harbison and Rex, 2010) because teachers who value PD typically work collaboratively and interactively in small groups toward their mutual professional growth.

### Teacher Characteristics

Teacher characteristics included gender (Jeong, 2006; Kim, 2009; Joo et al., 2013; Fang et al., 2017; Kang et al., 2018), age (Jeong, 2006; Fang et al., 2017), years of experience (Kim, 2009; Fang et al., 2017; Kang et al., 2018), administrative duty (Jeong, 2006; Lee and Hur, 2008; Kim, 2009; Joo et al., 2013), self-efficacy (Kim, 2009; Kim and Kim, 2009; Joo et al., 2013; Kim et al., 2015a; Park and Park, 2017; Lee and Kim, 2018; Lee et al., 2018) and perceptions about students (Kim, 2009). While self-efficacy and perceptions about students were generally positive and statistically significant to job satisfaction, the statistical significances of the other teacher characteristics were inconsistent in previous research. Some studies indicated that female teachers (Jung, 2016; Kang et al., 2018), younger teachers (Jeong, 2006), those with fewer years of teaching (Lee and Hur, 2008; Kim, 2009) or those with administrative duties (Jeong, 2006; Lee and Hur, 2008; Kim, 2009) were more satisfied with their jobs. However, factors such as gender, age, years of experience, and administrative duties were not statistically significant in other studies (Jeong, 2006; Lee and Hur, 2008; Kim, 2009; Joo et al., 2013; Fang et al., 2017; Kang et al., 2018).

### School Characteristics

#### School Demographics

School characteristics comprised *school demographics* and *school climate*. The demographic indicators investigated included school size, level, and type. Teachers working at larger schools (Cho et al., 2015; Kang et al., 2018) or at lower-level schools (Jung, 2016; Lee and Kim, 2018) expressed higher job satisfaction than those working at smaller or higher-level schools. Teachers at private schools typically had higher job satisfaction (Kim, 2009; Wi, 2013; Kim et al., 2016), while public school teachers reported higher job satisfaction in a study by Lee and Kim (2018).

#### School Climate

Variables related to school climate were grouped into *principal leadership* and *teacher cooperation*. Principal leadership was a strong indicator of job satisfaction. Specifically, transformational leadership (Lee, 2010, 2015; Cho et al., 2015; Lee et al., 2018), distributed leadership (Kim et al., 2016), and democratic leadership approaches (Lee, 2007) motivated or encouraged teachers to participate in school decision-making, which in turn improved teacher job satisfaction. Likewise, teacher empowerment was positively related to job satisfaction (Lee and Hur, 2008; Jung, 2009).

Teacher cooperation was identified as one of the crucial factors affecting job satisfaction. Teachers maintained higher job satisfaction in schools where there was consensus on the school’s objectives (Wi, 2013), there was a sound sense of community (Wi, 2013; Kim and Lee, 2014), or the climate encouraged PD and learning organization activities (Jung, 2009; Kim, 2009; Kim et al., 2015b; Fang et al., 2017; Kang et al., 2018; Lee et al., 2018). In particular, teacher cooperation had a statistically significant and positive effect on job satisfaction only for those who were actively involved in the professional learning community (Kim et al., 2018). In previous studies based on the TALIS data, teachers in caring school climates reported higher job satisfaction (Fang et al., 2017; Park and Park, 2017).

### Literature Summary

School characteristics generally appear to have a stronger relationship with teacher job satisfaction than teacher characteristics. With the exception of self-efficacy, other teacher characteristics such as gender, age, and years of experience did not indicate consistent relationships with job satisfaction. On the other hand, school characteristics regarding principal leadership, teacher cooperation, and school demographics were all found to be statistically significant to job satisfaction. Likewise, a meta-analysis reported that principal leadership affected job satisfaction more than the recorded teacher demographics, including gender, years of experience, and education level (Joo et al., 2013).

Previous studies have investigated teacher and school variables related to teacher job satisfaction, but only a limited number of variables were typically analyzed per individual study using conventional statistical methods. Regarding teacher characteristics, the practice of analyzing a small subset of variables from possible multicollinear data led to inconsistent results in the size and direction of coefficient estimates. TALIS provides hundreds of variables based on the responses of thousands of teachers and hundreds of principals in each participating country. The need to explore all possible TALIS variables in one statistical model in order to identify important job satisfaction predictors naturally emerged. To do this effectively, a machine-learning technique was the most appropriate tool.

## Group Mnet

The current study employed group Mnet as the machine-learning technique for selecting important variables. Group Mnet is categorized as *penalized regression* among machine-learning techniques. The idea of penalized regression is to introduce slight bias in the estimates, thus lowering variances and ultimately leading to reduction in mean squared errors (MSEs) and prediction errors. Penalized regression imposes a penalty on the objective function and diminishes some of the coefficient estimates. The earliest penalized regression was *ridge*. The original purpose of ridge was to handle multicollinearity problems in regression with least squares. Ridge adds a penalty to the diagonal elements of a singular *X^T^X* matrix to make the matrix invertible (Hoerl and Kennard, 1970), but ridge does not perform variable selection. Invented by Tibshirani (1996), the least absolute selection and shrinkage operator (LASSO) is one of the first and most popular penalized regression methods for selecting important variables. LASSO can also handle the so-called large *p* (number of variables), small *n* (number of observations) or high-dimensional data. LASSO uses a convex penalty that increases linearly regardless of the coefficient size, and its estimates are known to be inconsistent with respect to variable selection (Fan and Li, 2001; Meinshausen and Bühlmann, 2006; Zhao and Yu, 2006; Zou, 2006; Huang et al., 2008).

In response to LASSO shortcomings, a variety of methods have been proposed, including adaptive LASSO (Zou, 2006), smoothly clipped absolute deviation (SCAD; Fan and Li, 2001), and minimax concave penalty (MCP; Zhang, 2010). Adaptive LASSO incorporates weights into the LASSO penalty in order to achieve consistency, but the additional weight calculation is more computationally expensive. Obtaining the initial weights has also been an issue, particularly with high-dimensional data (Huang et al., 2008), which lead to variations of adaptive LASSO (e.g., Algamal and Lee, 2015; Algamal et al., 2015). While LASSO and adaptive LASSO utilize a convex penalty function, the penalties of SCAD and MCP are concave. Their concave penalties taper off with larger coefficients in absolute values, thereby yielding nearly consistent coefficient estimates (Fan and Li, 2001; Zhang, 2010). Compared to SCAD, MCP applies less shrinkage to non-zero coefficients, thus yielding less-biased coefficient estimates (Zhang, 2010; Breheny, 2016), and MCP is simpler to use (Huang et al., 2016).

Mnet in this study is a combination of MCP and ridge (Huang et al., 2016). The relationship between MCP and Mnet is the same as that of LASSO and elastic net. As LASSO does not perform well when correlations among variables are high, Zou and Hastie (2005) proposed elastic net, that is, a combination of LASSO and ridge. Elastic net selects variables due to the LASSO component and handles multicollinearity due to the ridge component. Likewise, Mnet retains the good features of both MCP and ridge, yields nearly consistent estimates, and effectively handles multicollinearity problems. Social science large-scale or panel data consisting of hundreds or thousands of variables cannot be entirely free from multicollinearity problems. Therefore, Mnet was chosen over MCP for the current study to analyze the TALIS data.

More specifically, group Mnet was employed in order to handle items in multiple-response categories. In a regression model, a categorical variable should be treated as a group after dummy coding. As a subgroup of regression, penalized regression also needs to treat dummy-coded variables from a categorical variable as a group. Such models are called *group* penalized regression. Therefore, *group* Mnet was the appropriate technique to handle both the categorical and continuous variables of TALIS.

Group MCP and group Mnet are explained by equations. Consider a linear regression model with *p* predictors. Suppose the predictors are divided into *K* non-overlapping groups, and the model can be written as in Eq. 1. *Y* is an *n* dimensional vector of a response variable. *X*_*k*_ is the *n*×*p*_*k*_ design matrix of the *p*_*k*_ predictors in the *k*-th group. β_*k*_ = (β_*k*,1_,…,β_*k*_,_*pk*_)*T* is the *p*_*k*_ dimensional vector of regression coefficients of the *k*-th group. ϵ is an *n* dimensional vector of mean zero.

(1) $$Y=\sum_{k=1}^{K}X_{k}\,\beta_{k}+\epsilon$$

The objective functions of group MCP and group Mnet for a Gaussian family are shown in Eqs 2 and 3, respectively. Group MCP and group Mnet are hereafter referred to as MCP and Mnet for brevity. The same first term in the right-hand side of the equations is the loss function of least squares. The same second term in the right-hand side of the equations is the MCP penalty. Notably, L_2_ norm (||β_*k*_||) is used in both MCP and Mnet to account for group membership of the variables. The regularization parameter of λ_1_ in Eqs 2 and 3 controls the amount of penalty. The γ parameter, the concavity penalty, regulates the penalization rate depending on the size of the coefficients. When the coefficients are larger than the product of the two penalties, the rate of the MCP penalty quickly drops, thereby applying less shrinkage to the coefficients and yielding less-biased estimates than LASSO. When the concavity penalty goes to infinity, the MCP penalty reverts back to the LASSO penalty. On top of MCP, Mnet adds the ridge penalty to the equation in order to handle multicollinearity, and the penalty parameter for ridge is λ_2_ (Eq. 3).

$${\hat{\beta }}^{M\,C\,P}=argmin_{\beta }[\frac{1}{2\,n}||Y-\sum_{k=1}^{K}X_{k}\beta_{k}|{|}^{2}$$

(2) $$+\sum_{k=1}^{K}J\left(||\beta_{k}|||\lambda_{1},\gamma \right)],$$

$$\text{where}\,J\,\left(x|\lambda_{1},\gamma \right)=\left\{ \begin{array}{cc} -\frac{1}{2\,\gamma }\,x^{2}+\lambda_{1}\,\left|x\right|, & \left|x\right|\leq \gamma \,\lambda_{1}\\ \frac{1}{2}\,\gamma \,\lambda_{1}^{2}, & \left|x\right|>\gamma \,\lambda_{1}. \end{array}\right.$$

$${\hat{\beta }}^{M\,n\,e\,t}=argmin_{\beta }[\frac{1}{2\,n}||Y-\sum_{k=1}^{K}X_{k}\beta_{k}|{|}^{2}$$

(3) $$+\sum_{k=1}^{K}J\left(||\beta_{k}|||\lambda_{1},\gamma \right)+\lambda_{2}\sum_{k=1}^{K}||\beta_{k}|{|}^{2}].$$

## Materials and Methods

### Data

A total of 2,933 middle school teachers and 177 principals participated in TALIS 2013 on behalf of Korea. Starting with 526 teacher variables and 393 principal variables of TALIS, those irrelevant to our analyses were removed, including ID, weighting, standardized scores and administration (e.g., IDTEACH, IDCNTRY, TCHWGT, TRWGT1, SCHWGT, SRWGT1, and IDLANG). Next, variables with 50% or higher missingness were deleted from further analyses. Categorical items from the TALIS questionnaires were dummy-coded.

Notably, a set of dummy-coded variables from the same item was treated as a group in the analysis, which was essentially the motivation of using group Mnet in this study. There were five such items: TT2G06, TT2G37, and TC2G06A to TC2G06C. For instance, TT2G06 (employment type), had three response categories: lifetime employment (1), more than 1 year term (2), and less than 1 year term (3). The original coding of this item was 1, 2, and 3, with the numbers only indicating categories. Therefore, the responses of 1, 2, and 3 were recoded as 0 or 1 with two dummy variables that were selected or removed as a group with group Mnet. TT2G37 asked teachers to select the subject they taught in 12 categories and was recoded with 11 dummy variables. Items TC2G06A to TC2G06C asked whether the principal had completed a school administration program (TC2G06A), a teacher training program (TC2G06B), or an instructional leadership training program or course (TC2G06C) either (1) before, (2) after, (3) before and after the principal took the position, or (4) never. Likewise, all three variables were coded with three dummy variables, respectively, and the set of dummy variables was treated as a group for analysis.

The teacher and principal datasets were merged after data cleaning. The final dataset consisted of 558 variables, including dummy-coded variables, of 2,577 middle school teachers and 165 principals. TALIS measures teacher job satisfaction with ten items of a 4-point Likert-like scale (TT2G46A to TT2G46J). The items included “The advantages of being a teacher clearly outweigh the disadvantages,” “If I could decide again, I would still choose to work as a teacher,” and “I am satisfied with my performance in this school.” The Cronbach alpha of the ten items was 0.88 with mean of 2.85 and standard deviation of 0.50. The mean of the ten items was the response variable of the current study, and the other 548 variables served as explanatory variable candidates.

### Missing Data Imputation

After data cleaning and merging, the k-nearest neighbors (k-NN) algorithm was employed to handle missing data. As a non-parametric method, k-NN has been one of the popular techniques for managing missing data in previous machine-learning literature (Troyanskaya et al., 2001). The idea of k-NN is straightforward. The k nearest neighbors or the k closest observations to a certain missing data point are identified in the multidimensional space. Depending on the type of variables, either majority voting or averaging is used to pinpoint the imputation value. Two things to consider when using k-NN are the value of k and the distance measure. The square root of the number of complete observations is typically used as the value of k (Beretta and Santaniello, 2016), and Gower distance is recommended for calculating distances of mixed-format data (Gower, 1971). This study used a Gower distance with 41 as the value of k calculated from 1,638 complete observations.

### Variable Selection

Of note, this study is one of the first to apply the idea of *relevance counts* (Shevade and Keerthi, 2003) and *stability selection* (Meinshausen and Bühlmann, 2010) to social science panel data with penalized regression. Both relevance counts and stability selection execute subsampling techniques for variable selection. Shevade and Keerthi (2003) illustrated relevance counts in gene selection with cancer data, a high-dimensional dataset. They repeated threefold cross validation (CV) 100 times at a penalty parameter, and counted how many times each predictor (gene) was selected in the 300 repetitions, which was named as a relevance count. Similarly, Meinshausen and Bühlmann (2010) proposed stability selection, also designed for high-dimensional data. Stability selection is obtained after data are perturbed many times and a cutoff is applied in a way to keep variables with a high selection probability in the model.

The steps for variable selection of this study were as follows. Firstly, the whole data were randomly divided with the ratio of 7:3 to get the training and test data, respectively. In the field of machine learning, data are split into training and test data. Then a model is built with training data, and the model is evaluated with test data. Secondly, using the training data, a 10-fold CV was executed with each value of the penalty parameter in range. As a result, the penalty parameter of the lowest MSE was identified, which lead to variable selection in that iteration. Thirdly, the same penalty parameter of the lowest MSE in the second step was applied to the test data, yielding the RMSE or prediction error of that iteration. This RMSE value was obtained for comparison and evaluation purposes of different penalized regression techniques. The three steps were repeated one hundred times with random seeds.

Particularly, the selection or non-selection of each variable from the second step was counted in the one hundred iterations, which served as the selection counts of the study. Both relevance counts and stability selection have been developed under the context of model validation with high-dimensional data. Previous studies with social science panel data have not yet considered the bias resulting from data-splitting in model validation. The current study repeated 100 times of modeling with random data-splitting, and presented variables selected 50% of the time or more. Variables selected at least once out of two runs were considered to be worth further investigation. All the programs were written in R 3.6.2. Specifically, the grpreg library (Breheny and Zeng, 2019) was used for penalized regression.

## Results

Table 1 summarizes the descriptive statistics of the 100 iterations of group Mnet as well as group MCP and group LASSO for comparison purposes. With regard to the number of selected variables, group LASSO tended to select the most variables, followed by group Mnet and group MCP, consistent with literature. Particularly, group LASSO almost doubled the number of group MCP. Group Mnet selected approximately 31.76 variables (SD = 9.67) on average, and the minimum, Q1, median, Q3, and maximum were 16, 25, 30, 39, and 59, respectively (Table 1). The RMSE of group Mnet ranged between 0.368 and 0.409, averaging at 0.393 (SD = 0.008). While the number of selected variables differed across penalized regression techniques, the RMSEs of them were similar in range, means, and SDs.

**TABLE 1:** Descriptive statistics of the number of selected variables and RMSE from 100 iterations of group Mnet, group MCP, and group LASSO.

	**# of selected variables**			**RMSE**		
	**Group**	**Group**	**Group**	**Group**	**Group**	**Group**
	**Mnet**	**MCP**	**LASSO**	**Mnet**	**MCP**	**LASSO**
Min	16.00	12.00	31.00	0.368	0.366	0.367
Q1	25.00	22.00	43.75	0.386	0.384	0.383
Median	30.00	28.50	55.00	0.393	0.392	0.388
Q3	39.00	33.25	67.25	0.399	0.397	0.395
Max	59.00	62.00	130.00	0.409	0.419	0.413
Mean	31.76	28.90	58.92	0.393	0.392	0.389
(SD)	(9.67)	(9.67)	(19.80)	(0.008)	(0.009)	(0.009)
						

Table 2 presents the relevance count results. While as many as 195 variables were selected at least once, only five variables were selected in all group Mnet runs. This result emphasizes the necessity to use selection counts in penalized regression. A total of 25 and 18 variables were selected 33 and 50% of the time, respectively. The current study presented variables selected at least once out of two runs for further interpretation, and there were 18 such variables, all from the teacher questionnaire.

**TABLE 2:** Selection counts from group Mnet.

	**# of selected variables**
≥1	195
≥33	25
≥50	18
≥95	8
=100	5

Teaching and Learning International Survey grouped the questionnaire items into 18 subsections, 11 from the teacher questionnaire, and 7 from the principal questionnaire. The selected 18 teacher variables were from 6 teacher subsections: background, PD, teacher feedback, teaching in general, your teaching, and school climate (Table 3). Only one item from background was selected; it asked the teachers the extent to which they felt prepared for the pedagogy of the subjects they taught (TT2G13B). As expected, the more prepared they felt, the higher their job satisfaction. Three items indicating barriers to PD were selected: a lack of employer support (TT2G27C), no time because of family responsibilities (TT2G27E), and no incentives for participating (TT2G27G). All three items were negatively related to teacher job satisfaction. In particular, the latter two items from the barriers subsection were selected 98 and 99 times, respectively.

**TABLE 3:** Variables selected after group Mnet and selection counts.

	**Variable**					
	**name**	**Subsection**	**Variable description**	**Mean (SD)**	**#**	**Response category**
1	TT2G13B	Background	Prepared for elements in teaching/pedagogy of the subject(s) I teach	0.052 (0.01)	70	{1, not at all; 2, somewhat; 3, well; 4, very well}
2	TT2G27C	Professional development	Barriers to professional development/there is a lack of employer support	−0.018 (0.015)	56	{1, strongly disagree; 2, disagree; 3, agree; 4, strongly agree}
3	TT2G27E	Professional development	Barriers to professional development/I do not have time because of family responsibilities	−0.03 (0.012)	98	
4	TT2G27G	Professional development	Barriers to professional development/there are no incentives for participating	−0.037 (0.016)	99	

5	TT2G30H	Teacher feedback	Has led to a positive change in/your classroom management practices	−0.039 (0.021)	59	{1, no positive change; 2, a small change; 3, a moderate change; 4, a large change}
6	TT2G30M	Teacher feedback	Has led to a positive change in/your job satisfaction	0.134 (0.011)	100	

7	TT2G31A	Teacher feedback	Agreement with/the best performing teachers in this school receive the greatest recognition	0.023 (0.014)	89	{1, strongly disagree; 2, disagree; 3, agree; 4, strongly agree}
8	TT2G31C	Teacher feedback	Agreement with/teacher appraisal and feedback are largely done to fulfill administrative requirements	−0.016 (0.011)	79	
9	TT2G32A	Teaching in general	Personal beliefs on teaching/my role as a teacher is to facilitate students’ own inquiry	0.018 (0.013)	59	

10	TT2G34E	Teaching in general	To what extend can you do the following/Motivate students who show low interest in school work	0.044 (0.02)	85	{1, not at all; 2, to some extent; 3, quite a bit; 4, a lot}
11	TT2G34K	Teaching in general	To what extend can you do the following/provide an alternative explanation	0.032 (0.021)	69	

12	TT2G41A	Your teaching	Agreement with statements/when the lesson begins, I wait quite a long time for students to quiet down	−0.034 (0.025)	53	{1, strongly disagree; 2, disagree; 3, agree; 4, strongly agree}
13	TT2G41B	Your teaching	Agreement with statements/students in this class take care to create a pleasant learning atmosphere	0.037 (0.017)	100	
14	TT2G41C	Your teaching	Agreement with statements/I lose quite a lot of time because of students interrupting the lesson	−0.08 (0.021)	98	
15	TT2G44A	School climate	Agreement with/this school provides staff with opportunities to participate in school decisions	0.088 (0.012)	100	
16	TT2G44E	School climate	Agreement with/there is a collaborative school culture which is characterized by mutual support	0.074 (0.02)	89	
17	TT2G45A	School climate	Agreement with what happens/in this school, teachers and students usually get on well with each other	0.209 (0.02)	100	
18	TT2G45B	School climate	Agreement with what happens/most teachers in this school believe that students’ well-being is important	0.055 (0.024)	100	

*Variables are presented in the order that they appear in the teacher questionnaire.*

Four items were selected from teacher feedback, two from outcomes of feedback (TT2G30) and the other two from perceptions of feedback and appraisal systems (TT2G31). Teachers tended to have higher job satisfaction when they felt that feedback from the school directly led to a larger positive change in job satisfaction (TT2G30M). This variable was selected in every run of group Mnet. The more the teachers agreed that the best performing teachers in their school received the greatest recognition, the more their job satisfaction increased (TT2G31A). The more they disagreed with the statement, “Teacher appraisal and feedback are largely performed only to fulfill administrative requirements,” the more their job satisfaction decreased (TT2G31C). These three items related to feedback were consistent with common sense. It was intriguing to find that the teachers who believed that feedback from the school directly led to less positive change in classroom management practices had higher job satisfaction (TT2G30H). This variable was selected about once out of two runs.

The subsections, *teaching in general* and *your teaching*, each had three items selected. Those who agreed more with the statement that their role as a teacher was to facilitate students’ own inquiry had higher job satisfaction (TT2G32A). Teachers who tried to motivate students who showed low interest in school work (TT2G34E) or offered alternative explanations when students were confused (TT2G34K) also had higher job satisfaction. Teachers who responded by saying that they waited a considerable length of time for students to quiet down at the beginning of lessons (TT2G41A) or sacrificed too much time when students interrupted lessons (TT2G41C) had lower job satisfaction. Conversely, those who agreed that “students in their class take care to create a pleasant learning atmosphere” (TT2G41B) had higher job satisfaction. This particular variable was selected one hundred times in one hundred runs.

Lastly, *school climate* had four items selected. Teachers who agreed more with the following statements had higher job satisfaction: “This school provides staff with opportunities to participate in school decisions” (TT2G44A), “There is a collaborative school culture characterized by mutual support” (TT2G44E), “In this school, teachers and students usually work well with each other” (TT2G45A), and “Most teachers in this school believe that students’ well-being is important” (TT2G45B). The first two, TT2G44A and TT2G44E, are items related to the participatory school climate, while TT2G45A and TT2G45B are items related to the caring nature of the school climate. In particular, the school climate items had a higher chance of being selected in the model relative to other items. All items regarding a caring school climate and one item regarding a participatory school climate (TT2G44A) were selected in every run, and the other item regarding a participatory school climate (TT2G44E) was selected 89 times out of 100.

## Discussion

A total of 548 variables from the teacher and principal questionnaires were explored after data cleaning and merging, and 18 variables were identified as important after selection counts. Among the 18 variables, 7 had been studied in previous research while the other 11 were newly identified.

### Variables Investigated in Previous Research

The seven variables investigated in previous research comprised two items from teacher self-efficacy, three regarding perceptions about students, and two related to a caring school climate. Teacher demographics, including gender, age, years of experience, and administrative duties were frequently investigated variables; however, they did not reveal any consistent relationship with job satisfaction, and the current study concluded that none of them were important. Although variables on principal leadership (Lee, 2007; Lee and Hur, 2008; Kim and Kim, 2009; Cho et al., 2015; Kim et al., 2015a) and school demographics (Cho et al., 2015; Kang et al., 2018) were statistically significant to job satisfaction, none of them were identified as important after selection counts in this study.

On the other hand, teacher self-efficacy and the classroom climate were selected as important. This is consistent with previous research (Kim, 2009; Kim and Kim, 2009; Joo et al., 2013; Kim et al., 2015b; Park and Park, 2017; Lee and Kim, 2018; Lee et al., 2018). In particular, TALIS 2013 measured teacher self-efficacy in three domains: classroom management, instruction, and student engagement, and there were four items in each domain. The two selected items out of these 12 were “Motivate students who show low interest in school work” (TT2G34E) and “Provide an alternative explanation” (TT2G34K). These represented student engagement and instruction, respectively. No item from classroom management was selected.

Three items were selected regarding classroom climate. Two of them, “When the lesson begins, I wait quite a long time for students to quiet down” (TT2G41A) and “I lose quite a lot of time because of students who interrupt lessons” (TT2G41C) were clear indications of disruptive classroom climates. Not surprisingly, both had negative relationships with job satisfaction. The other item, “Students in this class take care to create a pleasant learning atmosphere” (TT2G41B) was indicative of a caring classroom climate and thus had a predictable positive relationship with job satisfaction. Likewise, two items on caring school climates were selected as important. These items, “In this school, teachers and students usually get on well with each other” (TT2G45A) and “Most teachers in this school believe that student well-being is important” (TT2G45B) were positively related to job satisfaction and consistent with Fang et al. (2017) and Park and Park (2017).

### Newly Found Variables

A total of 11 variables were newly found and identified as important after selection counts. They were items related to preparedness for the pedagogy of the subject, barriers to PD, perceptions on feedback, and participatory school climates. Many of the variables appear to be unique items to TALIS and thus were not investigated in previous research.

To begin, job satisfaction was positively related to teachers’ perceptions about their preparedness for the pedagogy of the subject they taught (TT2G13B). Teacher preparation programs typically consist of courses on content knowledge (CK), pedagogical content knowledge (PCK), and pedagogical knowledge (PK). In a similar context, TALIS asked teachers the degree to which they felt prepared for the content, pedagogy, and classroom practice of the subject they taught (TT2G13A, TT2G13B, and TT2G13C). Only the pedagogy related to the subject was selected as an important predictor of job satisfaction. This emphasizes the importance of stressing PCK in teacher preparation and accreditation programs. In-service teachers should also have opportunities to update their PK, including material related to current technological advances and curriculum changes that may help maintain their job satisfaction.

Barriers to PD are one of the unique sets of factors presented by TALIS, and they have rarely been asked in other questionnaires. Among the seven items regarding barriers to PD, three were selected: “no time because of family responsibilities” (TT2G27E), “no incentives for participating” (TT2G27G), and “lack of employer support” (TT2G27C). All three hinder teachers’ PD participation. Family responsibilities of teachers are personal matters, but incentives for participation and employer support are something schools and districts can improve on their end. In particular, financial support significantly increased Korean teachers’ PD participation (Song and Park, 2014). When schools and districts devise plans to boost teacher PD participation, monetary support should be considered one of the most powerful incentives. Ultimately, this will have a positive effect on job satisfaction.

Variables on teacher feedback are also one of the distinguishing features of TALIS. Questions 28–31 under the teacher feedback subsection in the TALIS 2013 questionnaire collected substantial information regarding the feedback received. Responses to the sub-items of the four major questions were converted to 69 variables for analysis after data cleaning, and four variables were selected as a result. Of special note, teachers who indicated that the feedback they received at school led to no positive changes in their classroom management had higher job satisfaction than those who reported positive changes (TT2G30H). For those who experienced difficulties managing their classrooms, they might have assumed that any feedback or advice was beneficial, thus assuaging their sense of insecurity as a teacher. On the contrary, teachers who indicated no positive effects of feedback on their classroom management practices might have felt that they had few classroom management issues to begin with. Consequently, their job satisfaction was higher than those with more serious classroom management issues. This hypothesis needs to be tested with empirical data in further studies. Other variables identified as important related to the appraisal system. Teachers generally had lower job satisfaction when they believed that appraisals and feedback were largely provided only to fulfill administrative requirements (TT2G31C). Satisfaction was also lower for those who agreed less with the statement that the best performing teachers received the greatest recognition (TT2G31A). Taken together, these findings suggest that principals and school district administrators must maintain the feedback and appraisal systems and they must be fair and meaningful to their teachers.

Finally, two items were drawn from participatory school climates whose relationships with job satisfaction had to this point remained uninvestigated. It was well-documented in previous research that principals who were more willing to share decision-making with teachers contributed to a more collaborative school culture and, therefore, higher teacher job satisfaction. The newly found items extended the subject of participation in decision-making from only teachers to incorporate staff in general (TT2G44A), and they emphasized the mutual support of all stakeholders, including staff, parents, and students as an indication of a collaborative school (TT2G44E). That is to say, a principal who shares decision-making with staff is likely to increase teacher job satisfaction, and teachers are more satisfied when the school culture is collaborative and encourages mutual support of all stakeholders. One thing to note is that the TT2G44A TALIS item measured teacher perceptions regarding the empowerment of staff in decision-making without differentiating the faculty (teachers) from office personnel. It would have been more informative to present separate items for teaching staff and office staff, respectively, and then study the relationship of teacher perceptions about principal leadership with job satisfaction.

### Suggestions and Future Research Topics

Despite efforts to clean the data, merge, and group, all 18 important predictors selected were from the teacher questionnaire, and no grouped items were identified after selection counts. This result alone may indicate that questions answered by principals as well as machine-learning techniques incorporating the grouping effect may not be needed to predict teacher job satisfaction. When the dependent variable is from the teacher questionnaire, questions answered by teachers tend to be reported as more statistically significant than those by principals. In particular, when using panel/cohort data such as TALIS, principal variables are likely to be less sensitive for detecting differences than teacher variables. Teachers are nested in schools and the single value of a principal variable is applied to all teachers in the same school after merging. Regarding the grouping effect, research based on social science data is quite limited; however, previous machine-learning research showed similar results. For instance, by employing group LASSO, Yoo and Rho (2017) identified 15 out of 338 variables for predicting middle school students’ life satisfaction, but no grouped variables were selected in that study. They did not investigate this issue, and further studies are warranted regarding the factors in variable section with machine-learning techniques that incorporate the grouping effect. The number of categories and the unequal sample sizes of the categories could be a good starting point.

Some of the TALIS questions might be revisited and modified to specific countries, if necessary. TALIS 2013 offered large-scale data of 34 jurisdictions worldwide that measured various aspects of educational practices with nearly equivalent standards. While researchers using TALIS data relish the advantages of comparing different jurisdictions, the standardization approach may sometimes yield problematic issues in actual practice. For instance, questions TC2G17 and TC2G18 capture perceptions of school management teams. However, Korean schools do not typically have school management teams and do not differentiate them from school governing boards. It is likely that principals had difficulties with these questions and spent unnecessary effort forming responses. This time could have been spent more meaningfully on questions that are more appropriate in the Korean context. Another international comparison study, TIMSS, allows country-specific variables in the questionnaires. For instance, TIMSS asks students if they have a computer, an Internet connection, and a study desk, among other factors, and it also leaves three or four items that can include country-specific indicators of wealth. Following the practice of TIMSS, it would be worthwhile for TALIS to include country-specific adaptability in the questionnaires.

## Conclusion

Job satisfaction of teachers relates to education outcomes and can eventually lead to national academic competitiveness (Jung, 2009). The current study identified and explored important predictors to teacher job satisfaction using TALIS data. As TALIS offers a variety of variables on teaching and learning that reflect the perspectives of teachers and principals, machine learning was the appropriate tool to explore hundreds of TALIS variables in one prediction model. Specifically, group Mnet was employed. Mnet executes variable selection with consistency and handles multicollinearity as a combination of MCP and ridge, while group Mnet treats dummy-coded variables from a categorical variable as a set in variable selection.

To summarize the results of the study, teachers and school administrators perceive their roles for increasing teacher job satisfaction as follows. Teachers should motivate students who show low interest in school work and provide alternative explanations based on the belief that their role as teachers is to facilitate student self-inquiry and well-being, both of which are important for improving learning outcomes. The family responsibilities of teachers should be alleviated whenever possible, and they should master the pedagogy of the subjects they teach. School principals and district leaders should encourage participatory and caring school climates. Teachers tend to be more satisfied with their jobs in schools where students help create pleasant learning environments, teachers and students work well together, and teachers perceive that the school culture is collaborative and mutually supportive. Principals also need to provide teachers with sufficient incentives for participating in ongoing PD and should not give the impression that teacher appraisals and feedback are largely performed only to fulfill administrative requirements. Instead, teachers should be assured that the best performing teachers in their school will always receive the greatest recognition.

## Data Availability Statement

The data that support the findings of this study are available in OECD at https://stats.oecd.org/index.aspx?datasetcode=talis_2013%20. These data were derived from the following resources available in the public domain: http://www.oecd.org.

## Ethics Statement

Ethical review and approval was not required for the study on human participants in accordance with the local legislation and institutional requirements as this study analyzed data from a public domain. Written informed consent for participation was not required for this study in accordance with the national legislation and the institutional requirements.

## Author Contributions

JY designed the study and wrote the manuscript. MR performed data analyses.

## Conflict of Interest

The authors declare that the research was conducted in the absence of any commercial or financial relationships that could be construed as a potential conflict of interest.
